# Targeting Cutaneous T-Cell Lymphoma Cells by Ingenol Mebutate (PEP005) Correlates with PKCδ Activation, ROS Induction as Well as Downregulation of XIAP and c-FLIP

**DOI:** 10.3390/cells10050987

**Published:** 2021-04-23

**Authors:** Uly Sumarni, Ulrich Reidel, Jürgen Eberle

**Affiliations:** Apoptosis Regulation in Skin Cancer, Skin Cancer Center Charité, Department of Dermatology Venerology und Allergology, Charité—Universitätsmedizin Berlin, Corporate Member of Freie Universität Berlin and Humboldt-Universität zu Berlin, 10117 Berlin, Germany; uly.sumarni@googlemail.com (U.S.); ulrich.reidel@charite.de (U.R.)

**Keywords:** cutaneous T-cell lymphoma (CTCL), PEP005, PKC-delta, apoptosis, caspases, ROS, XIAP, c-FLIP

## Abstract

New therapeutic strategies are needed for cutaneous T-cell lymphoma (CTCL), and the plant extract ingenol mebutate (PEP005) may be considered. PEP005 has been approved for actinic keratosis, and proapoptotic activities were described in different cancer cells. Here, we aimed to investigate its efficacy in four CTCL cell lines and its mode of action. While HuT-78 and HH responded with induced apoptosis as well as with loss of cell viability and cell proliferation, MyLa and SeAx remained resistant. Interestingly, both sensitive and resistant cells showed caspase-8 activation and enhanced levels of reactive oxygen species (ROS), while final caspase-3 activation was restricted to sensitive cells. Apoptosis induction was prevented by the caspase inhibitor QVD-Oph as well as by the antioxidant vitamin E. Caspase activation by PEP005 may be explained to some extent by the downregulation of the caspase antagonistic proteins c-FLIP and XIAP in sensitive cells, whereas both proteins were strongly expressed in resistant cells. Finally, PEP005 resulted in the activation of proapoptotic PKCδ, and the PKC inhibitor bisindolylmaleimide I reduced apoptosis, caspase-3 processing and ROS production, as well as restored cell viability. In conclusion, PKCδ appeared as a central player in apoptosis regulation in CTCL cells, also suggesting its therapeutic targeting.

## 1. Introduction

Cutaneous T-cell lymphomas (CTCL) form a heterogeneous group of extranodal non-Hodgkin’s lymphomas and are characterized by primary cutaneous manifestation and clonal proliferation of skin-homing memory T-lymphocytes. In clinical appearance and prognosis, CTCL are clearly distinct from the histotypically cognate systemic lymphomas and their possible secondary cutaneous manifestations. The different characteristics of CTCL were acknowledged by the WHO/EORTC classification. The group of CTCL encloses Mycosis fungoides, Sézary syndrome and CD30(+) lymphoproliferative disorders [1,2]. The incidence of CTCL is at 3–4 cases per one million per year in Europe and at 10 cases per one million per year in the United States [3,4]. In its early stage, CTCL may show an often indolent clinical course, whereas in the later phase, it frequently transforms to a rapidly growing, malignant phenotype, significantly decreasing life expectancy [2,5]. New and alternative therapeutic options are needed for early and late disease.

The elimination of tumor cells by the induction of apoptosis represents a principal goal in cancer therapy, while therapy resistance is frequently explained by apoptosis deficiency [6,7]. Also several treatments of CTCL were related to apoptosis induction in tumor T-cells, e.g., phototherapy, photopheresis, the retinoid bexarotene and the histone deacetylase inhibitor vorinostat [8,9,10,11].

Two major pathways drive apoptosis induction. Thus, extrinsic proapoptotic pathways are initiated by death ligands, such as CD95L/FasL and TNF-related apoptosis-inducing ligand (TRAIL). Their binding to death receptors results in the formation of a death-inducing signaling complex and activation of initiator caspase-8 and caspase-10 [12]. Caspase-8 can cleave and activate the main effector caspase-3, which itself cleaves a large number of death substrates with the final result of DNA fragmentation and apoptosis induction [13]. While caspase-8 activation can be inhibited in a competitive mechanism by cellular FLICE-like inhibitory protein (c-FLIP) [14,15], caspase-3 is negatively regulated through the binding of chromosome X-linked inhibitor of apoptosis protein (XIAP) [16,17]. On the other hand, intrinsic apoptosis pathways are activated in response to cellular stress situations, e.g., by chemotherapy or DNA damage, and are critically regulated by the family of Bcl-2 proteins [18]. In particular, they rely on the loss of mitochondrial membrane potential and release of mitochondrial factors, such as cytochrome c, which triggers the activation of initiator caspase-9, which again can activate effector caspases [19].

In CTCL cells, activation of the extrinsic apoptosis pathways is of major importance. Thus, apoptosis resistance correlated with reduced expression of death receptor CD95/Fas as well as with high and constitutive expression of c-FLIP [20,21,22]. Additionally, activation of the prosurvival transcription factor NF-κB and of STAT3 was reported [23,24,25,26]. In particular, different therapeutic strategies, such as NSAIDs, suberoylanilide hydroxamic (SAHA) pentoxifylline and indirubin derivatives, resulted in the downregulation of c-FLIP and XIAP in CTCL cells [10,27,28,29]. Furthermore, reactive oxygen species (ROS) may contribute to the regulation of apoptosis, as shown in CTCL cells for an indirubin derivative [27]. ROS may derive from mitochondrial leakage or other sources [30], but their relation to described apoptosis pathways is less clear to date.

The protein kinase C (PKC) family of isoenzymes encloses several serine-threonine kinases, which are involved in the regulation of different cellular processes, including cell proliferation, cell differentiation and apoptosis [31]. While PKCα and PKCβ in particular support cell proliferation and cell invasion [32], PKCδ was reported as proapoptotic. Following possible phosphorylation and translocation steps, PKCδ can be activated through processing, which releases the active catalytic domain (41 kDa) from its 78 kDa proform. It was suggested that PKCδ can induce apoptosis through tyrosine phosphorylation and activation of caspase-3 [33,34].

Ingenol 3-angelate or ingenol mebutate (PEP005) is a hydrophobic diterpene ester isolated from the plant Euphorbia peplus. Its antineoplastic effects have been reported in different kinds of cancer cells, such as in leukemia, colon cancer and melanoma cells [31,35,36,37,38]. PEP005 has been approved by the FDA for the treatment of actinic keratosis in 2012. Here, we investigated its effects on cutaneous T-cell lymphoma cells and elucidated its mechanism of action. In this way, we aimed to identify strategies and additional molecular targets in CTCL cells for the induction of apoptosis.

## 2. Materials and Methods

### 2.1. Cell Culture and Treatment

For investigating the effects of PEP005 in CTCL cells, four representative CTCL cell lines were used. MyLa derived from a plaque biopsy of a patient with MF [39]; SeAx [40] and HuT-78 [41] derived from PBMCs of patients with Sézary syndrome; HH (ATCC, Manassas, VA, USA; CRL2105) derived from peripheral blood of a patient with aggressive CTCL [42]. Cells were maintained at 5% CO_2_ in RPMI 1640 growth medium (Life Technologies, Darmstadt, Germany) supplemented with 10% FCS, 600 μM L-glutamine and antibiotics.

Most assays were performed in 24-well plates, and 5 × 10^4^ cells were seeded per well. Cells were treated with 2–2000 nM ingenol mebutate (PEP005; AdipoGen Life Sciences, Liestal, Switzerland). Control cells received the solvent DMSO in the same concentrations as used for PEP005-treated cells (max. 0.2%). For caspase inhibition, cells received the pan-caspase inhibitor QVD-Oph (Abcam, Cambridge, UK; 10 µM) at 1 h before agonists were applied. For ROS scavenging, cells were pre-treated for 1 h with 1 mM α-tocopherol (vitamin E, Fluka, Steinheim, Germany). For inhibition of PKCδ, bisindolylmaleimide I (Bis 1; Cayman Chemical, Ann Arbor, MI, USA) was used at 1 µM in HH and at 0.25 µM in HuT-78, respectively.

### 2.2. Determination of Apoptosis, Cytotoxicity, Cell Viability and Cell Proliferation

Quantification of apoptosis was performed by cell cycle analysis. Harvested cells were lysed in hypotonic buffer, and isolated nuclei were stained for 1 h with 40 mg/mL propidium iodide (Sigma-Aldrich, St. Louis, MO, USA). Cells in G1, G2 and S-phase as well as sub-G1 cells were quantified by flow cytometry at FL3A with a FACS Calibur (BD Bioscience, Bedford, MA, USA). Due to the washing out of small DNA fragments, nuclei with less DNA than G1 (sub-G1) correspond to apoptotic cells.

Cell viability was determined by staining cells with calcein-AM (PromoCell, Heidelberg, Germany), which is converted through intracellular esterase activity in viable cells to green-fluorescent calcein. Cells, grown and treated in 24-well plates, were harvested and stained for 1 h with 0.5 µM calcein-AM at 37 °C. After labeling, cells were washed with PBS and measured by flow cytometry (FL2H).

Cell proliferation was determined by WST-1 assay (Roche Diagnostics) following the protocol of the supplier. The assay depends on the cleavage of the water-soluble tetrazolium (WST) salt by mitochondrial dehydrogenases in metabolically active cells.

### 2.3. Mitochondrial Membrane Potential and Reactive Oxygen Species (ROS)

Mitochondrial membrane potential was determined by staining cells with the fluorescent dye TMRM^+^ (Sigma-Aldrich). Cells, grown and treated in 24-well plates, were harvested and stained for 20 min at 37 °C in 1 µM TMRM^+^. After washing twice with PBS, cell staining was quantified by flow cytometry (FL2H).

For the determination of intracellular ROS levels, cells grown in 24-well plates were pre-incubated for 1 h with the fluorescent dye H_2_DCFDA (D-399, Thermo Fisher Scientific, Hennigsdorf, Germany, 10 µM), before starting treatment with effectors. After 2–24 h treatment, cells were harvested, washed several times with PBS and analyzed by flow cytometry (FL1H). As positive controls, cells were treated with H_2_O_2_ (1 mM, 1 h).

### 2.4. Western Blotting

For Western blotting, total protein extracts were obtained in cell lysis buffer containing 150 mM NaCl, 1 mM EDTA, 1% NP-40, 50 mM Tris (pH 8.0), as well as phosphatase and protease inhibitors. Following SDS polyacrylamide gel electrophoresis, proteins were blotted on nitrocellulose membranes.

Primary antibodies of Cell Signaling (Danvers, MA, USA): caspase-3 (9662, rabbit, 1:1000), cleaved caspase-3 (9664, rabbit, 1:1000), caspase-8 (9746, mouse, 1:1000), caspase-9 (9502, rabbit, 1:1000), XIAP (2042, rabbit, 1:1000). Primary antibodies of Santa Cruz Biotech (Dallas, TX, USA): c-FLIP (sc-5276, mouse, 1:500), p21 (sc-6246, mouse, 1:500), p53 (sc-126, mouse, 1:500), GAPDH (sc-32233, mouse, 1:1000). The antibody for PKCδ (PA587443, rabbit polyclonal, 1:1000) was from Thermo Fisher Scientific (Hennigsdorf, Germany). As secondary antibodies, peroxidase-labelled goat anti-rabbit and goat anti-mouse (Dako, Hamburg, Germany; 1:5000) were used.

### 2.5. Statistical Analyses

All assays were performed in triplicate determinations, and at least two independent experiments were performed. Presented Western blot data were verified by at least two independent series of cellular extracts. Statistical significance was proven by a Student’s *t*-test (2-tailed, heteroscedastic) using all data of independent experiments (at least six individual measurements); *p*-values < 0.05 were considered as statistically significant.

## 3. Results

### 3.1. Effects of PEP005 on Apoptosis, Cell Viability and Cell Proliferation

**Figure 1:**
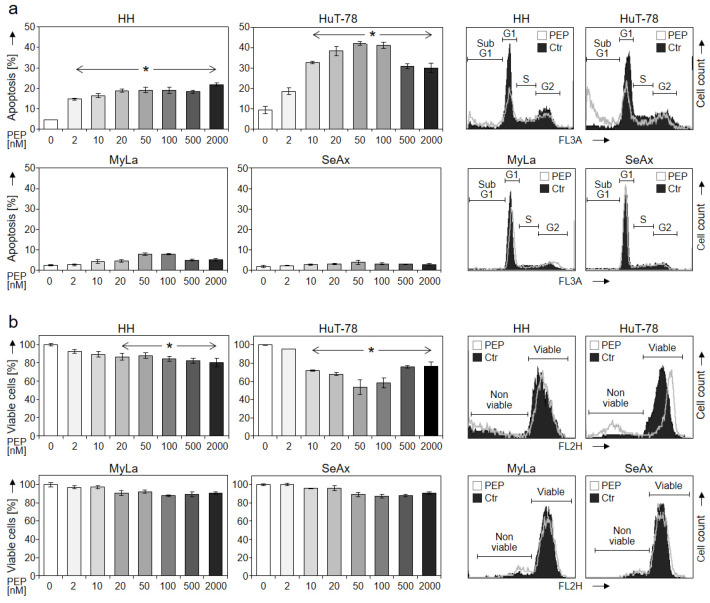
Dose dependency of induced apoptosis and decreased cell viability. Apoptotic rates (propidiumiodide staining, **a**) and cell viability (calcein staining, **b**) were determined in four CTCL cell lines (HH, HuT-78, MyLa and SeAx) treated for 48 h with increasing concentrations of PEP005 (2, 10, 20, 50, 100, 500, 2000 nM). Apoptotic rates correspond to percentages of cells with fragmented DNA, which were determined as sub-G1 cells. Cell viability values are shown in relation to non-treated controls, which were set to 100%. Characteristic histograms of cells treated with 50 nM PEP005 (PEP) are shown on the right side in overlays with control cells (Ctr). Cell populations in cell cycle phases G1, S, G2 and sub-G1 cells as well as viable and non-viable cells are indicated in (**a**,**b**), respectively. Mean values of triplicates ± SDs of representative experiments are shown. At least two independent experiments (each one with triplicates) revealed highly similar data. Statistical significance was calculated from all values (at least 6) and is shown for treated cells vs. controls (* *p* < 0.05).

Induction of apoptosis and loss of cell viability represent highly critical issues in cancer therapy. For investigating the potential therapeutic efficiency of PEP005 in cutaneous T-cell lymphoma, four CTCL cell lines (HH, HuT-78, MyLa and SeAx) were treated with increasing concentrations (2 nM–2 µM) for 48 h. Apoptosis was determined by cell cycle analysis and quantification of sub-G1 cells, while cell viability was monitored by calcein-AM staining. Apoptosis was significantly induced in HH and HuT-78, resulting in 19% and 42% apoptotic cells, respectively (50 nM PEP005). There was no further increase in apoptosis with higher concentrations; rather, 50 nM appeared as most efficient. In clear contrast, almost no apoptosis induction was observed in MyLa and SeAx (Figure 1a). In parallel with apoptosis, cell viability significantly decreased in sensitive cells, resulting in 88% of the control (HH, 50 nM) and 54% of the control (HuT-78, 50 nM), respectively. Again, effects were less pronounced in MyLa and SeAx (Figure 1b).

**Figure 2:**
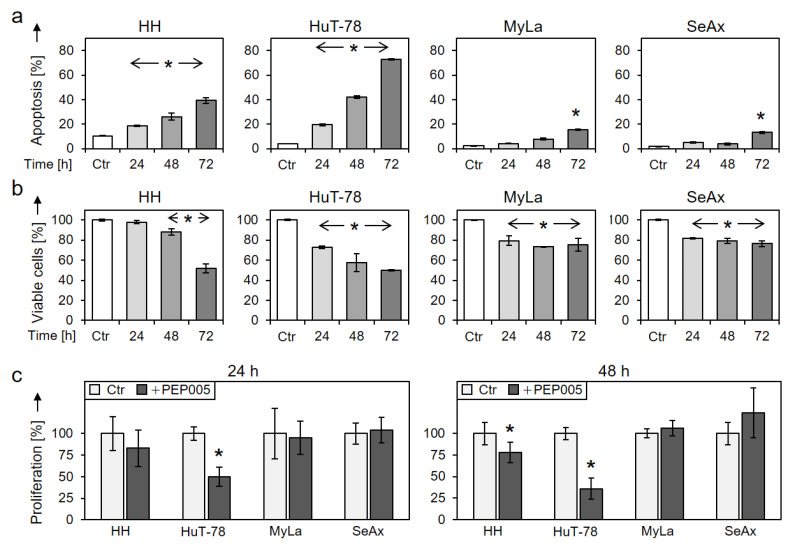
Time dependency and cell proliferation. Time dependency of (**a**) induced apoptosis and of (**b**) reduced cell viability were determined in four cell lines at 24, 48 and 72 h of treatment with 50 nM PEP005. For cell viability, non-treated controls were set to 100% (Ctr). (**c**) Cell proliferation rates of CTCL cell lines at 24 and 48 h in response to 50 nM PEP005 were determined by WST-1 assay. Values are given in relation to non-treated controls (Ctr), set to 100%. At least two independent experiments were performed, each one with triplicate values; statistical significance was calculated from all values (* *p* < 0.05).

Time dependency of apoptosis and cell viability were investigated at 24, 48 and 72 h (50 nM PEP005). The rate of apoptotic cells further increased with time resulting in 39% and 73% at 72 h in HH and HuT-78, respectively (Figure 2a). In parallel, cell viability further decreased with time, resulting in 52% and 50% viable cells at 72 h in HH and HuT-78, respectively, as compared to the controls (Figure 2b). Also some less pronounced effects were seen in resistant cells at 72 h. Thus, apoptosis was at 15% and 13%, and cell viability was at 75% and 76% in MyLa and SeAx, respectively (Figure 2a,b).

In parallel, cell proliferation decreased in HH and HuT-78, as determined by WST-1 assay. Thus, at 48 h, cell proliferation in response to 50 nM PEP005 was at 78% and 36% in HH and HuT-78, respectively, whereas no antiproliferative effects were seen in MyLa and SeAx (Figure 2c).

### 3.2. Effects on Mitochondrial Membrane Potential (MMP) and ROS Levels

**Figure 3:**
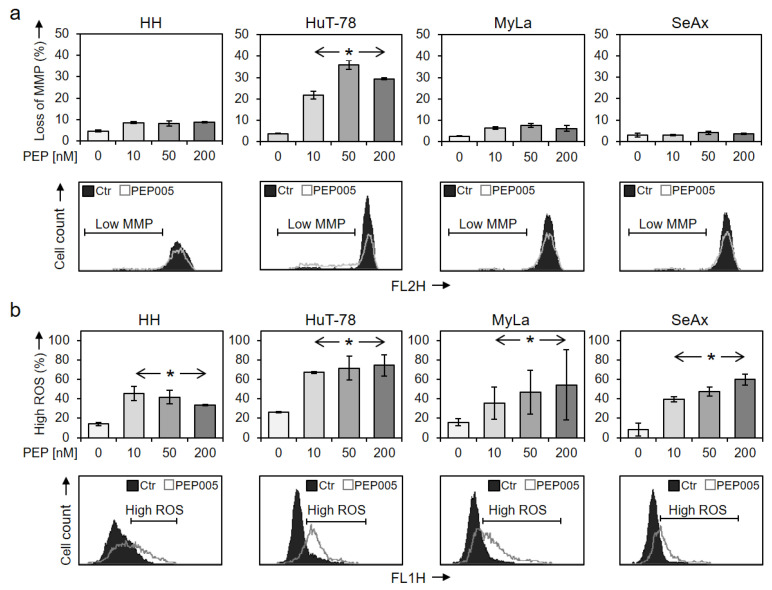
Loss of mitochondrial membrane potential and enhanced ROS levels. Changes of (**a**) mitochondrial membrane potential (MMP) and of (**b**) ROS levels were determined at 24 h of treatment with 10, 50 and 200 nM PEP005. Mean values of triplicates ± SDs are shown; a second independent series of experiments revealed highly comparable results. Representative histograms (overlays of treated cells vs. controls) are given below the bar charts, and cells with low MMP (**a**) or with high ROS (**b**) are indicated. Statistical significance was calculated from two independent experiments (at least six values) and is indicated for treated cells vs. controls (* *p* < 0.05).

Loss of MMP, which is characteristic of the activation of intrinsic apoptosis pathways, was monitored at 24 h of treatment with 50 nM PEP005. In the most responsive cell line HuT-78, a significant loss of MMP was seen (36% of cells), while HH, MyLa and SeAx were not responsive (Figure 3a). Cell death pathways in cancer cells may be further induced by enhanced levels of reactive oxygen species (ROS). Indeed, increased ROS levels in response to PEP005 were seen in all four cell lines at 24 h of treatment, which, however, did not correlate with the apoptotic response nor with the concentration applied (Figure 3b).

### 3.3. Effects of PEP005 on Apoptosis-Related Proteins

**Figure 4:**
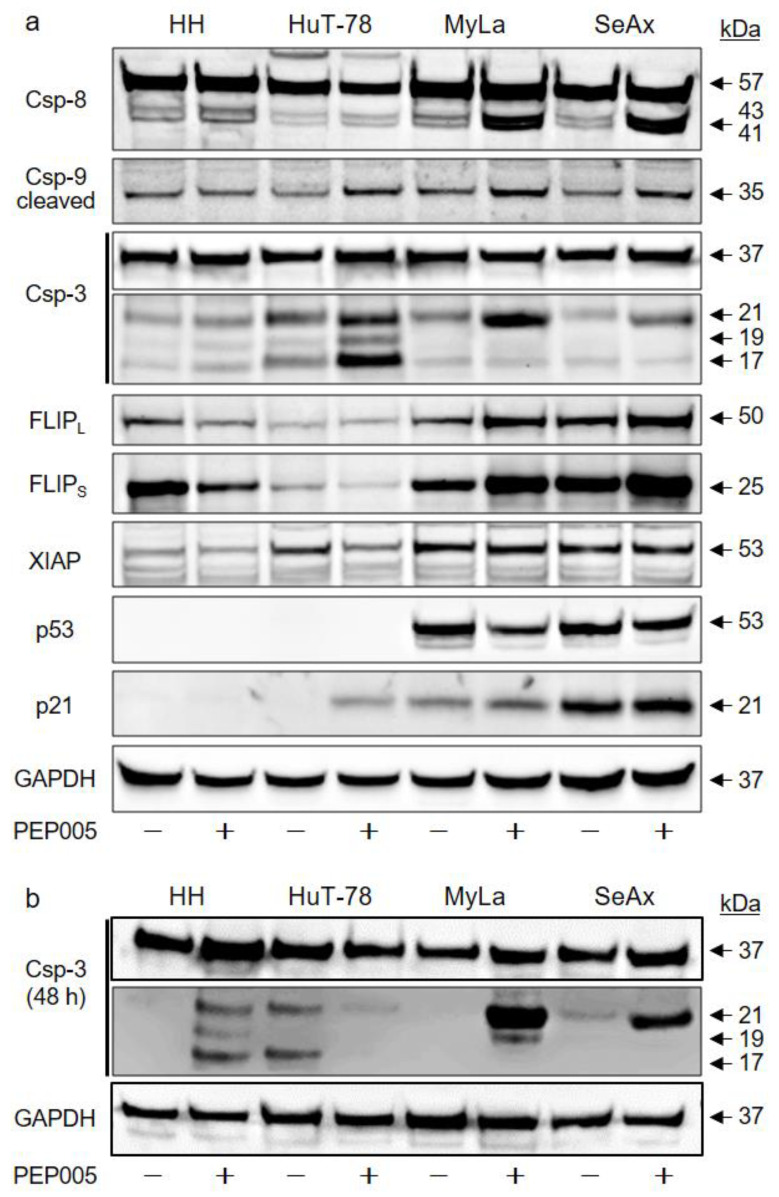
Effects on protein expression. (**a**) Effects of PEP005 (50 nM) on caspases and other apoptosis-related proteins were determined by Western blotting in four CTCL cell lines at 24 h of treatment. (**b**) For caspase-3, 48 h treatment was also investigated. (**a**,**b**) An amount of 30 µg of each protein extract was loaded per lane. Blots were probed with antibodies for caspase-8 (proform, 57 kDa; cleavage products, 43, 41 kDa), caspase-9 (cleavage product, 35 kDa), caspase-3 (proform, 35 kDa; cleavage products, 21, 19, 17 kDa), c-FLIP long/short isoforms (c-FLIP_L/S_, 50/25 kDa), XIAP (53 kDa), p53 and p21 (53 and 21 kDa, respectively). The housekeeping protein GAPDH (37 kDa) was used as the loading control. Two independent series of protein extracts and Western blotting experiments revealed highly comparable results.

To illuminate the mechanisms of PEP005-induced apoptosis in CTCL cells, apoptosis-related proteins were investigated by Western blotting. Processing of the initiator caspase of the extrinsic apoptosis pathway (caspase-8; 43, 41 kDa fragments) and of the initiator caspase of the intrinsic pathway (caspase-9, 35 kDa fragment) are indicative of an initiation of proapoptotic caspase cascades. Interestingly, strongest caspase-8 processing was seen in the two resistant cell lines MyLa and SeAx. Additionally, some caspase-9 processing was seen in HuT-78, MyLa and SeAx (Figure 4a). Initiator caspases thus appeared to be activated in response to PEP005 also in resistant CTCL cells, suggesting that apoptosis pathways may be blocked at subsequent steps.

The activation steps of effector caspases are different. As for caspase-3, an intermediate fragment of 21 kDa, which is due to processing by initiator caspases, does not yet represent an activated caspase-3. The mature caspase is rather represented by a 17 kDa fragment, which is due to caspase-3 autoprocessing. In line with caspase-8 activation in the resistant cells MyLa and SeAx, caspase-3 was also processed in these cells to its 21 kDa fragment; however, final processing to the active caspase-3 (17 kDa) was lacking in resistant cells at 24 and 48 h (Figure 4a,b). In clear contrast, the 17 kDa fragment of caspase-3 was strongly induced in HuT-78 at 24 h (Figure 4a) and in HH at 48 h (Figure 4b).

We thus looked for factors that can suppress caspase activity. Striking differences were observed for c-FLIP long and short isoforms (c-FLIP_L/S_) and for XIAP, which serve as caspase-8 and caspase-3 antagonists, respectively. In resistant MyLa and SeAx, these proteins were strongly expressed, and c-FLIP_L/S_ were even upregulated by PEP005. In contrast, c-FLIP_L/S_ were only weakly expressed in HuT-78, and they were downregulated in HH by PEP005. Similarly, XIAP was weakly expressed in HH and downregulated by PEP005 in HuT-78 (Figure 4a). These findings suggest a critical role of caspase antagonistic factors for limiting the sensitivity of CTCL cells to PEP005.

The expression of the tumor suppressor and proapoptotic transcription factor p53 negatively correlated with PEP005 sensitivity, as it was strongly expressed in resistant cells but completely lacking in HH and HuT-78. Similarly, the cell cycle inhibitor p21 was completely lacking in HH but strongly expressed in MyLa and SeAx. Only in HuT-78, p21 followed the expected regulation, namely, it was induced by PEP005 in parallel with inhibited cell proliferation (Figure 4a). Thus, p21 may contribute to the inhibition of cell proliferation in HuT-78 but not in other cell lines.

### 3.4. Inhibition of Apoptosis by QVD and by Vitamin E

**Figure 5:**
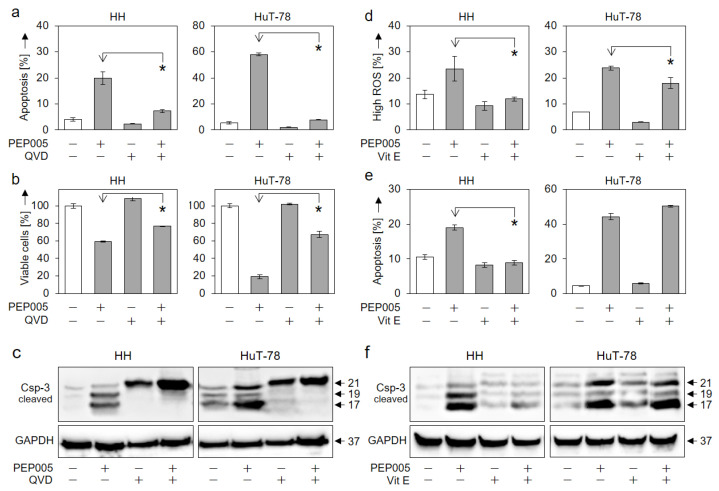
Inhibition of PEP005-mediated effects by QVD-Oph and vitamin E. Antagonistic effects of the pan-caspase inhibitor QVD-Oph (QVD, 5 µM, **a**–**c**) and of the antioxidant vitamin E (VitE, 1 mM, **d**–**f**) were determined in HH and HuT-78 cells. Assays included the determination of apoptosis (**a**,**e**, 48 h), cell viability (**b**, 48 h), ROS production (**d**, 24 h) and caspase-3 processing (**c**,**f**, Western blots, 24 h). (**a**,**b**,**d**,**e**) Mean values of triplicates ± SDs of representative experiments are shown. At least two independent experiments were performed. Statistical significance was calculated by using all values (at least 6) and is indicated for combination treatments vs. PEP005 alone (* *p* < 0.05). (**c**,**f**) Caspase-3 cleavage products (21, 19, 17 kDa) are indicated. Each 30 µg of protein extracts were loaded per lane. GAPDH (37 kDa) served as loading control. Two independent series of protein extracts and Western blots revealed highly comparable results.

We aimed to prove the caspase dependency of PEP005-induced apoptosis as well as its relation to ROS production. By using the pancaspase inhibitor QVD-Oph, apoptosis induction in HH and HuT-78 was almost completely prevented (Figure 5a), which was associated with the partial restoration of cell viability (Figure 5b). Western blotting revealed that caspase-3 processing in response to PEP005 was arrested by QVD-Oph at the level of the p21 intermediate cleavage product, clearly indicating the inhibition of the caspase-3 auto-processing step through QVD-Oph (Figure 5c).

Concerning the role of ROS, we show here that the antioxidant vitamin E can abolish ROS production in response to PEP005 in HH cells, while ROS could only be slightly decreased in HuT-78 (Figure 5d). In parallel, vitamin E prevented PEP005-induced apoptosis (Figure 5e) as well as the activation of caspase-3 in HH but not in HuT-78 (Figure 5f). We thus conclude that the antineoplastic effects of PEP005 in CTCL cells enclosed both caspase and ROS-mediated pathways.

### 3.5. Role of PKCδ in PEP005-Induced Apoptosis in CTCL

**Figure 6:**
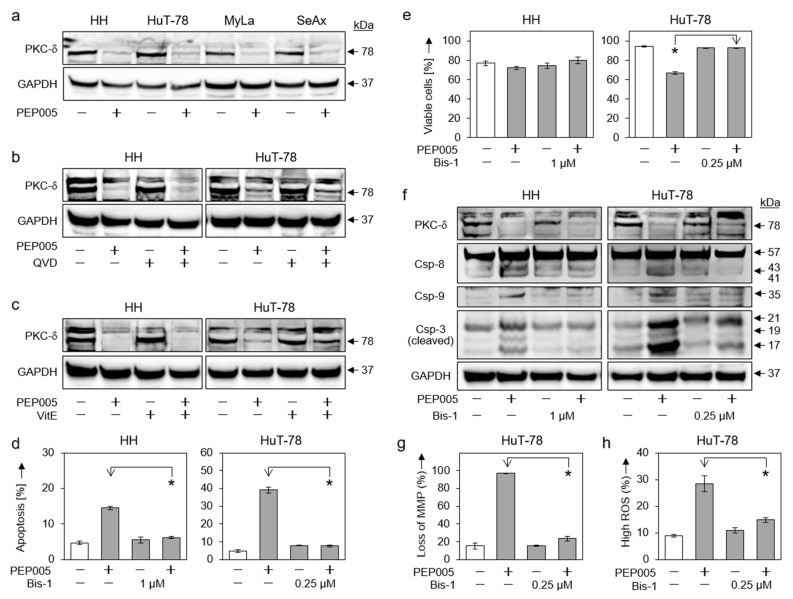
Role of PKCδ in PEP005-induced apoptosis. (**a**) Effects of PEP005 (50 nM, 24 h) on PKCδ proform (78 kDa) were investigated in four CTCL cell lines. (**b**,**c**) Lacking effects of QVD-Oph (QVD, 5 µM, (**b**)) and vitamin E (VitE, 1 mM, (**c**)) on PEP005-induced downregulation of PKCδ proform are shown (50 nM, 24 h). (**d**,**e**) Inhibition of PEP005-induced apoptosis (**d**) and restoration of cell viability (**e**) by Bis-1 in HH and HuT-78. Cells were treated for 24 h with PEP005 (50 nM) and/or Bis-1 (HH, 1 µM; HuT-78, 0.25 µM). (**f**) Inhibition of PEP005-mediated caspase-3, -8, and -9 processing through Bis-1, as investigated by Western blotting in HH and HuT-78. Cells were treated for 24 h with 50 nM PEP005; Bis-1 was used at 1 (HH) and 0.25 µM (HuT-78), respectively). (**g**,**h**) Antagonistic effects of Bis-1 on PEP005-mediated loss of MMP (**g**) and on PEP005-induced ROS production (**h**) in cell line HuT-78 (Time: 24 h; PEP005: 50 nM; Bis-1: 0.25 µM). (**a**–**c**,**f**) For Western blotting, 30 µg of each protein extract was loaded per lane, and blots were probed with antibodies for PKCδ proform (78 kDa), cleaved caspase-3 (21, 19, 17 kDa), caspase-8 (proform, 57 kDa; cleavage products, 43/41 kDa) and caspase-9 (cleavage product, 35 kDa). GAPDH (37 kDa) was used as loading control. For Western blots, two independent series of protein extracts revealed highly comparable results. (**d**,**e**,**g**,**h**) Mean values of triplicates ± SDs of representative experiments are shown. At least two independent experiments showed highly comparable results. Statistical significance was calculated from all values (at least 6) and is indicated for combination-treated cells vs. PEP005-treated cells (* *p* < 0.05).

Proapoptotic activities have been attributed to protein kinase C delta (PKCδ), and previous studies have indicated that PEP005 functions as a PKCδ inhibitor. Here, we show significant activation of PKCδ in all four CTCL cell lines in response to 50 nM PEP005 (Figure 6a). Thus, in all cell lines, the 78 kDa proform was strongly reduced, indicating its processing, which was described to release the 41 kDa active catalytic domain [33,34]. PKCδ activation appeared to be upstream of both caspase-3 and ROS, as its processing could be inhibited neither by QVD-Oph nor by vitamin E (Figure 6b,c).

**Figure 7:**
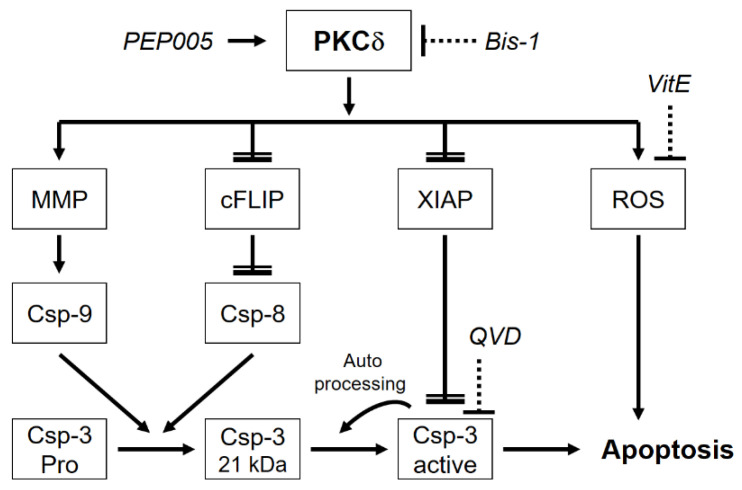
Molecular mechanisms of PKCδ-induced apoptosis in CTCL cells. In CTCL cells, PKCδ is induced by PEP005 and drives loss of mitochondrial membrane potential (MMP) related to caspase-9 activation, downregulation of c-FLIP related to caspase-8 activation, downregulation of XIAP related to caspase-3 activation as well as induction of reactive oxygen species (ROS) related to additional apoptosis promotion. The proform of caspase-3 (Pro) is cleaved by caspase-9 and caspase-8 to a 21 kDa intermediate product. Final processing to active caspase-3 is due to caspase-3 autocatalytic activity. For understanding the mechanisms, the PKC inhibitor bisindolylmaleimide I (Bis-1), the antioxidant vitamin E (VitE) and the pancaspase inhibitor QVD-Oph (QVD) were used. Further explanations are provided in the text.

The role of PKCδ in PEP005-mediated effects in CTCL cells was further investigated by the PKC inhibitor bisindolylmaleimide-1 (Bis-1). Underlining the critical role of PKCδ, PEP005-induced apoptosis was completely abolished by Bis-1 in HH and HuT-78, and cell viability was restored (Figure 6d,e). While Bis-1 did not affect PKCδ activation itself, it largely abolished PEP005-mediated processing of caspase-3, -8 and -9 (Figure 6f). Bis-1 also prevented PEP005-mediated loss of MMP (Figure 6g) and ROS production (Figure 6h) in HuT-78 cells. Thus, PKCδ appeared to be a master regulator in PEP005-induced effects in CTCL cells, and its activation appeared to be upstream of all other effects (Figure 7).

## 4. Discussion

The plant extract ingenol mebutate PEP005 is considered for clinical development in blood and solid tumors. Its antineoplastic effects have been reported in cells of different cancer types, such as leukemia, colon cancer and melanoma [31,35,36,37,38,43]. In the present study, significant induction of apoptosis in response to PEP005 is reported in two of four CTCL cell lines, associated with reduced cell viability and cell proliferation. The sensitivity did not correlate to the cells’ origin of either MF or Sézary patients [44]; rather, cell lines from both groups were either sensitive (HH, HuT-78) or resistant (MyLa, SeAx).

Deficient apoptosis programs are of particular importance for the pathogenesis of cancer, and thus induction of apoptosis and decrease in cell viability represent particular goals in cancer therapy [7]. Proapoptotic effects of PEP005 have also been reported previously in leukemia, colon cancer and melanoma cell lines [31,35,37,43]. A selective activity of PEP005 in cancer cells was suggested, as it did not inhibit the growth of human neonatal fibroblasts [38] and did not induce apoptosis in normal CD34(+) cord blood myeloblasts [35]. Despite several side effects reported for PEP005, the results of clinical trials and finally its FDA approval may largely rule out severe effects on normal human T-lymphocytes [45,46].

A major challenge in cancer therapy is to reach effective drug concentrations in target cells. For HH and HuT-78, relatively moderate concentrations of only 50 nM turned out to be highly effective, and apoptosis induction was not further increased by increasing the dose. Comparable effective concentrations (10–50 nM) were reported for sensitive myeloid leukemia cells and melanoma cells as well as for one of four tested colon cancer cell lines (Colo205). In contrast, several other melanoma and colon cancer cell lines were largely resistant or needed concentrations of 1–100 µM [31,35,37,38,43]. Also MyLa and SeAx remained resistant at 48 h up to 2 µM of PEP005. Thus, treatment with PEP005 may apply for a subset of CTCL patients, and there seems to be an optimal concentration range for obtaining the best response, which is low enough that it may also be achieved in clinical settings.

Apoptosis can be mediated by caspase-dependent or -independent pathways [47]. Activation of the main effector caspase-3 in response to PEP005 was reported in melanoma cells at a concentration of 500 nM [37], but the question, where in the pathway the caspase cascade may be blocked in resistant cells has not been raised so far. Indicating the activation of caspase cascades, caspase-3 was processed in HuT-78 at 24 h and in HH at 48 h to its 17 kDa mature cleavage product, whereas its processing was arrested in resistant MyLa and SeAx at a 21 kDa intermediate, non-active cleavage product. The situation was different for initiator caspase-8, which appeared as strongly processed and activated in resistant cells. This can be explained by a described mechanism, according to which caspase-8 mediates the processing of procaspase-3 up to the 21 kDa intermediate product, whereas further processing from 21 to 17 kDa is due to caspase-3 auto-processing [48] (Figure 7). As caspase-3 activity was lacking in resistant cells, its processing was arrested at 21 kDa. The essential role of caspases was proven by a pan-caspase inhibitor, which almost completely abolished apoptosis in sensitive cells.

The interruption of the caspase cascade can be due to caspase-antagonistic proteins. While caspase-8 activation can be inhibited in a competitive way by the short and long c-FLIP isoforms [14,15], caspase-3 activity is inhibited by XIAP [16,17]. We report here characteristic correlations of PEP005 sensitivity with the expression and regulation of XIAP and c-FLIP. Thus, in the sensitive CTCL cell lines, both XIAP and c-FLIP were either weakly expressed, or they were downregulated by PEP005. In contrast, in resistant cells, they were strongly expressed and not downregulated. It is suggestive that the constitutively high expression of XIAP in resistant cells prevents caspase-3 activity and activation. The critical function of caspase antagonists in CTCL cells was also described in response to other treatments. Thus, c-FLIP was downregulated by SAHA and NSAIDs, while XIAP was downregulated by SAHA and pentoxifylline in CTCL cells [10,28,29].

Loss of mitochondrial membrane potential (MMP) is part of intrinsic apoptosis pathways activated in response to cellular stress situations [19]. Loss of MMP in response to PEP005 was reported both for sensitive and resistant melanoma cells, when using concentrations of >100 µM [43]. In CTCL cells, the loss of MMP was apparently not absolutely required for PEP005-mediated effects, as not seen in HH. However, the additional activation of intrinsic apoptosis pathways may have a contributory role and may explain the particularly high sensitivity of HuT-78.

An increasing body of evidence in recent years has shown that reactive oxygen species (ROS) may result in induction of apoptosis in cancer cells [27,47,49,50]. Indeed, ROS were induced by PEP005 in all four CTCL cell lines, and antioxidative treatment by vitamin E decreased ROS levels in HH, which was further associated with a reduction in apoptosis and restoration of cell viability. In this setting, ROS may serve as an additional promoter of apoptosis, but ROS alone were not sufficient for the induction of apoptosis in resistant cells.

Mutations in the tumor suppressor p53 were reported in CTCL and associated with advanced disease stage as well as poor prognosis [51]. P53 mutations were also reported in the cell lines used here, as in the coding region (HuT-78, SeAx), a splicing mutation (HH) and gene duplications (MyLa, HH) [44]. Only in MyLa cells, p53 appeared to be transcriptionally active [52]. Here, we saw a complete lack of basal p53 protein expression in HuT-78 and HH, as also reported previously [52]. In colon cancer cells, PEP005-mediated effects on cell cycle arrest and apoptosis induction were p53-independent [53]. This is in agreement with our results, as apoptosis was induced in HH and HuT-78, despite p53 mutation and lack of expression.

Induction of PEP005-induced apoptosis in sensitive CTCL cells was accompanied by a reciprocal loss of cell proliferation. The cyclin-dependent kinase inhibitor p21 can either be activated by p53 or p53-independent [54]. Here, we found significant upregulation of p21 in HuT-78 in response to PEP005, which may contribute to the high sensitivity of this cell line. Clearly, the regulation of p21 in these cells must be p53-independent.

PEP005 was described as an activator of PKCδ, and the dominant role of PKCδ in PEP005-mediated effects was demonstrated in leukemia, colon cancer and melanoma cells [31,35,38,43]. The proapoptotic function of PKCδ has also been shown in response to DNA damaging agents, UV radiation, phorbol 12-myristate-12-acetate and ROS. PKCδ is activated through the processing of its 78 kDa proform, which releases the active 41 kDa catalytic domain [33,34]. Also in the CTCL cells reported here, activated PKCδ turned out to be the central player in PEP005-induced apoptosis. Its activation was shown here by the consistent loss of the 78 kDa proform, as antibodies did not detect the 41 kDa fragment.

In smooth muscle cells, it was suggested that PKCδ may act both upstream and downstream of caspase-3. Thus, PKCδ could also be processed and activated through caspase-3 [34]. This explanation was ruled out here for CTCL cells, as PKCδ was also activated by PEP005 in resistant MyLa and SeAx, while caspase-3 was not active, and, furthermore, PKCδ processing was not abolished by the pancaspase inhibitor QVD-Oph. Thus, other proteases must be involved in the processing of PKCδ in CTCL cells. PKCδ activation was also not controlled by induced ROS, as its activation was not impaired by the antioxidant vitamin E.

In conclusion, we show here in an in vitro approach the efficacy of PEP005 in a subset of CTCL cell lines, and we shed more light on its mode of action. To further approach a clinical development, the investigation of ex vivo patient samples and in vivo animal experiments may be the next steps. Providing a comparable situation in patients’ tumor T-cells, a major challenge will be to identify patients that could profit from this strategy. Based on our in vitro data, resistant cell lines were characterized by distinct molecular characteristics, such as p53 and p21 expression, as well as by enhanced expression of c-FLIP and XIAP, which may serve as candidates for the stratification of patients.

Concerning the mode of action, PKCδ appeared to be an upstream player in PEP005-induced effects, as also proven by the PKC inhibitor Bis-1. An upstream and thus non-specific activity of this inhibitor was largely ruled out, as it remained without effect on PKCδ processing itself. Clearly proving the high-ranking role of PKCδ in PEP005-mediated effects in CTCL cells, Bis-1 prevented all downstream effects as apoptosis induction, caspase-3 activation, induction of ROS, loss of MMP and cell viability. These findings result in the perception that activated PKCδ represents a master regulator in apoptosis control in CTCL cells, triggering the loss of MMP; induction of ROS; downregulation of c-FLIP and XIAP; and the activation of caspase-9, caspase-8 and caspase-3, which may all be linked in a logical network, as suggested in Figure 7. PKCδ thus appears to be an additional molecular target in CTCL for the induction of apoptosis, which may further suggest its therapeutic targeting by PEP005 or by other related strategies.

## Data Availability

Not applicable.
