# Correction: Sumarni et al. Targeting Cutaneous T-Cell Lymphoma Cells by Ingenol Mebutate (PEP005) Correlates with PKCδ Activation, ROS Induction as Well as Downregulation of XIAP and c-FLIP. *Cells* 2021, *10*, 987

**DOI:** 10.3390/cells14070535

**Published:** 2025-04-03

**Authors:** Uly Sumarni, Ulrich Reidel, Jürgen Eberle

**Affiliations:** Apoptosis Regulation in Skin Cancer, Skin Cancer Center Charité, Department of Dermatology Venerology und Allergology, Charité—Universitätsmedizin Berlin, Corporate Member of Freie Universität Berlin and Humboldt-Universität zu Berlin, 10117 Berlin, Germany; uly.sumarni@googlemail.com (U.S.); ulrich.reidel@charite.de (U.R.)

In the original publication [1], there was a mistake in Figure 6 as published. The GAPDH panels in Figures 5f and 6b,f were the same due to the accidental swapping of the blots. The corrected Figure 6 appears below. The authors state that the scientific conclusions are unaffected. This correction was approved by the Academic Editor. The original publication has also been updated.

## Figures and Tables

**Figure 6:**
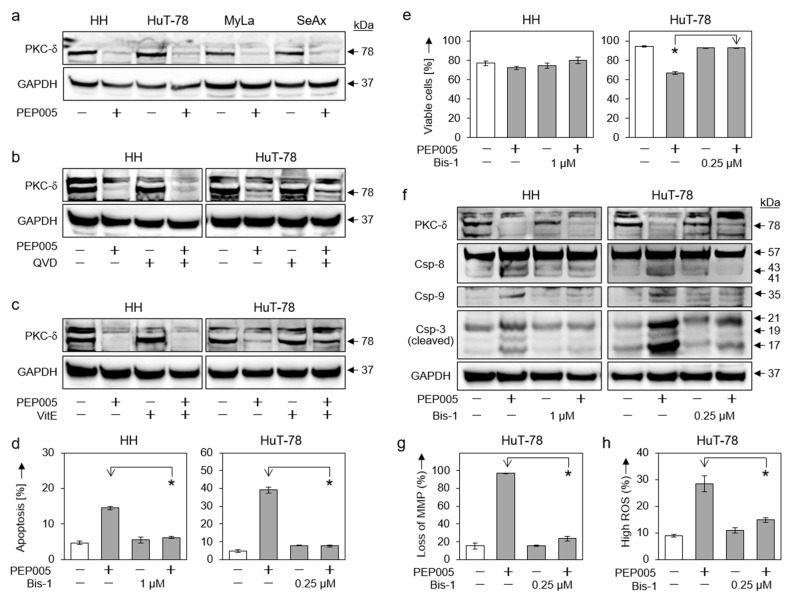
Role of PKCδ in PEP005-induced apoptosis. (**a**) Effects of PEP005 (50 nM, 24 h) on PKCδ proform (78 kDa) were investigated in four CTCL cell lines. (**b**,**c**) Lacking effects of QVD-Oph (QVD, 5 µM, (**b**)) and vitamin E (VitE, 1 mM, (**c**)) on PEP005-induced downregulation of PKCδ proform are shown (50 nM, 24 h). (**d**,**e**) Inhibition of PEP005-induced apoptosis (**d**) and restoration of cell viability (**e**) by Bis-1 in HH and HuT-78. Cells were treated for 24 h with PEP005 (50 nM) and/or Bis-1 (HH, 1 µM; HuT-78, 0.25 µM). (**f**) Inhibition of PEP005-mediated caspase-3, -8, and -9 processing through Bis-1, as investigated by Western blotting in HH and HuT-78. Cells were treated for 24 h with 50 nM PEP005; Bis-1 was used at 1 (HH) and 0.25 µM (HuT-78), respectively). (**g**,**h**) Antagonistic effects of Bis-1 on PEP005-mediated loss of MMP (**g**) and on PEP005-induced ROS production (**h**) in cell line HuT-78 (Time: 24 h; PEP005: 50 nM; Bis-1: 0.25 µM). (**a**–**c**,**f**) For Western blotting, 30 µg of each protein extract was loaded per lane, and blots were probed with antibodies for PKCδ proform (78 kDa), cleaved caspase-3 (21, 19, 17 kDa), caspase-8 (proform, 57 kDa; cleavage products, 43/41 kDa) and caspase-9 (cleavage product, 35 kDa). GAPDH (37 kDa) was used as loading control. For Western blots, two independent series of protein extracts revealed highly comparable results. (**d**,**e**,**g**,**h**) Mean values of triplicates ± SDs of representative experiments are shown. At least two independent experiments showed highly comparable results. Statistical significance was calculated from all values (at least 6) and is indicated for combination-treated cells vs. PEP005-treated cells (* *p* < 0.05).
